# Public procurement of antineoplastic agents used for treating breast cancer in Brazil between 2013 and 2019

**DOI:** 10.1186/s12885-022-09851-3

**Published:** 2022-07-15

**Authors:** Ranailla Lima Bandeira dos Santos, Vera Lúcia Edais Pepe, Claudia Garcia Serpa Osorio-de-Castro

**Affiliations:** 1 Sergio Arouca National School of Public Health/Oswaldo Cruz Foundation, Rio de Janeiro, Brazil; 2 Department of Health Planning and Administration, Sergio Arouca National School of Public Health/Oswaldo Cruz Foundation, Rio de Janeiro, Brazil; 3 Department of Pharmaceutical Policies and Pharmaceutical Services, Sergio Arouca National School of Public Health/Oswaldo Cruz Foundation, Rio de Janeiro, Brazil

**Keywords:** Pharmaceutical services, Drug costs, Antineoplastic agents, Federal government, Breast neoplasms

## Abstract

**Background:**

Breast cancer is the most common cancer among women in Brazil and the country’s public health care system is the main care provider. Timely treatment can increase the chance of cure, prevent metastasis and improve quality of life. Effective public procurement of antineoplastic agents can therefore improve access to drug therapy. This study investigates patterns in the procurement of selected antineoplastic agents used for treating breast cancer by public bodies and avoidable expenditure on these drugs between January 2013 and December 2019.

**Methods:**

We selected antineoplastic agents used for adjuvant or preoperative chemotherapy listed in the 2018 Breast Cancer Diagnosis and Treatment Guidelines and included in category L of the WHO Anatomical Therapeutic Chemical classification system. We analyzed regular purchases of antineoplastic agents registered in the Integrated General Services Administration System (SIASG), considering purchased quantity, unit price, date of purchase and procuring entity. Prices were inflation-adjusted to July 2019 based on the National Consumer Price Index.

**Results:**

A total of 10 antineoplastic agents were selected. Trastuzumab and tamoxifen accounted for the largest share of total spending and largest volume of purchases, respectively. The Ministry of Education was the largest purchaser in volume terms of all the drugs studied, except trastuzumab 440 mg, where the category “Other Institutions” accounted for most purchases, and vinorelbine 20 mg, where the Ministry of Health made most purchases. The category “Other Institutions” accounted for the largest share of total spending. Total avoidable expenditure was R$99,130,645. Prices paid for medicines and avoidable expenditure were highest in the Ministry of Defense.

**Conclusions:**

The differences observed in the performance of different categories of buyers as to amounts purchased and prices practiced for antineoplastic agents could be reduced by employing strategies to expand the centralization of purchases, resulting in expanded access to breast cancer medicines in the public sector.

## Background

Cancer is a major public health problem in both developing and developed countries. Globally, the disease is the second leading cause of death and was responsible for around 9.6 million deaths in 2018. One in five deaths is due to cancer and approximately 70% of cancer deaths occur in low and middle-income countries [1]. In Brazil, neoplasms are considered a public health problem due to their significant disease burden, high care costs and complex health care needs, which include surgery, chemotherapy, radiotherapy and complementary tests. Data from the country’s mortality information system (SIM, acronym in Portuguese) reveal that there were 235,301 cancer deaths in 2019, with the largest number of deaths occurring in the Southeast region [2].

Breast cancer has the highest mortality rate among malignant neoplasms in Brazil and worldwide. The World Health Organization (WHO) estimates that there are more than one million new cases of this type of cancer worldwide each year, making it the most common cancer among women [3]. The population-adjusted breast cancer mortality rate is increasing and breast cancer is currently the leading cause of cancer deaths among women in Brazil, resulting in 13.68 deaths/100,000 population in 2015 [4]. Figures from the National Cancer Institute (INCA, acronym in Portuguese) show that there was an annual average of 66,280 new breast cancer cases in Brazil between 2020 and 2022, confirming that it is the most common cancer among women [5].

Breast cancer treatments include chemotherapy, which is used in between 60 and 70% of patients [6]. The steady rise in the cost of treatment using antineoplastic agents is worrying, especially considering that this class of medicines has a major impact on spending by the country’s national health service – *Sistema Único de Saúde* (SUS) or Unified Health System – accounting for 46% of total expenditure on medicines in 2012 [7, 8]. Spending on cancer treatment has risen dramatically in recent years, from R$470 million in 1999 to R$3.3 billion in 2015. Around two-thirds of this expenditure was related to chemotherapy [9]. In view of the high incidence and prevalence of breast cancer in the country and the import role the public health system plays in cancer treatment, the analysis of government procurement of medicines can provide essential information for understanding the availability of and access to antineoplastic agents.

The aim of this study was to analyze patterns in the procurement of selected antineoplastic agents used for treating breast cancer by public bodies between 2013 and 2019, focusing on purchase quantities, prices paid, and avoidable expenditure on these drugs.

## Methods

### Study Design

We conducted a quantitative cross-sectional study of public procurement of antineoplastic agents used for treating breast cancer between January 2013 and December 2019.

### Selection of antineoplastic drugs

The selection of antineoplastic drugs was based on those used for adjuvant (prophylactic) or preoperative (neoadjuvant/cytoreductive) chemotherapy listed in the 2018 Breast Cancer Diagnosis and Treatment Guidelines [10] and included in category L of the WHO Anatomical Therapeutic Chemical (ATC) classification system [11]. Only medicines purchased by the Ministry of Health in at least five of the seven years of the study period were included. Most of the drugs are only employed in breast cancer, while others have broader indications.

Substances used in combination therapy regimens were excluded (CEF – cyclophosphamide, epirubicin, 5-fluorouracil; CAF – cyclophosphamide, doxorubicin, 5-fluorouracil; AC - doxorubicin (adriamycin), cyclophosphamide; CMF – cyclophosphamide, methotrexate, 5-fluorouracil; and DC – docetaxel, cyclophosphamide).

The final sample included: anastrozole (1 mg); docetaxel (40 mg); exemestane (25 mg); letrozole (2.5 mg); paclitaxel (6 mg); tamoxifen (20 mg); trastuzumab (440 mg); and vinorelbine (10, 20 and 30 mg).^1^

### Data extraction

Data on purchase quantities and prices paid for the selected medicines were obtained from the Integrated General Services Administration System (SIASG). Run by the Ministry of Planning, Budgeting and Management, the data produced by this public procurement and contracting tool are publicly accessible [12]. All purchases made by the Ministry of Health’s Department of Health Logistics and by Ministry of Health hospitals and outpatient facilities, as well those made by university hospitals linked to the Ministry of Education, must be recorded in this system. Ministry of Defense medical services and state and municipal health services register purchases on their own systems and transfer the data to the SIASG [13]. Under Brazilian legislation, quality is assessed as part of the bidding procedures and for the purposes of this study, only active purchases made using competitive bidding procedures were included.

### Analysis

The following purchase characteristics were analyzed: medicine specification; unit/dosage form (tablet, capsule, ampoule); purchase date; purchase status (active or inactive); procurement entity; number of units purchased; unit price; and type of procurement (competitive bidding/normal; waiver of competitive bidding; or bidding not required). Furthermore, purchaser categories were assigned: Ministry of Health, Ministry of Education, Ministry of Defense, and “Other Institutions” (other government bodies and subnational organizations, including state and municipal health authorities).

As docetaxel, paclitaxel, trastuzumab and vinorelbine do not have a listed defined daily dose (DDD), for the purposes of this study purchase volume was standardized to mg to allow comparison between medicines. Volume was calculated by multiplying the total number of purchased dosage forms by the dose (mg) of each form.

The annual weighted average price per mg (WAP/mg) paid by each purchaser category for each medicine was calculated by multiplying the volume of each individual drug purchase by the unit price paid and dividing overall expenditure by the total number of mg purchased. We also calculated corrected WAP/mg to allow comparisons over time. Prices were inflation-adjusted to July 2019 based on annual variations in the National Consumer Price Index (IPCA), obtained using the Central Bank citizen’s calculator, available at https://www3.bcb.gov.br/CALCIDADAO. This method was used because the law regulating the pharmaceutical industry (Law 10,742/2003) applies an inflation-based cap to drug prices based on this index [14].

For each year, total spending on each medicine by each purchaser category was divided by the lowest WAP/mg for the medicine in the respective year and multiplied by the WAP/mg actually paid by the procuring entity to calculate how much would have been spent if the medicine had been purchased at the lower price. The resulting amount was then subtracted from actual expenditure to calculate “avoidable expenditure”.

Avoidable expenditure was then divided by the lowest WAP/mg for the medicine in the respective year to calculate the additional quantity of drugs that could have been purchased by applying the principle of economy. The purchase data were organized by year in separate spreadsheets, tabulated and analyzed in dynamic tables using Microsoft Excel® version 2205. The datasets generated during the current study are available in the Arca Dados repository, 10.35078/PPYTKP [15].

## Results 

### Purchases volumes

The Ministry of Education was the largest purchaser in volume terms of all the drugs studied, except trastuzumab 440 mg, where the category “Other Institutions” represented 41.29% of purchases, and vinorelbine 20 mg, where the Ministry of Health accounted for 41.24% of purchases. The Ministry of Health was the second largest purchaser of all drugs, except exemestane 25 mg and letrozole 2.5 mg. The Ministry of Defense was the second largest purchaser of the latter drugs, accounting for 18.96 and 28.86% of purchases, respectively. Tamoxifen 20 mg accounted for the largest volume of purchases across all purchaser categories (Table 1).

**Table 1:** Public procurement of antineoplastic agents used for treating breast cancer - purchase quantities in mg by purchaser category. Brazil, 2013–2019

Medicine	Ministry of Education		Ministry of Health		Ministry of Defense		Other Institutions		Total (mg)
	N	%	N	%	N	%	N	%	
Anastrozole 1 mg^a^	10,838,515	41.25	8,803,262	33.50	2,913,665	11.09	3,722,340	14.17	**26,277,782**
Docetaxel 40 mg^b^	12,097,460	45.50	8,352,420	31.41	1,834,800	6.90	4,304,760	16.19	**26,589,440**
Exemestane 25 mg^c^	21,923,725	48.42	7,926,800	17.51	8,587,175	18.96	6,842,850	15.11	**45,280,550**
Letrozole 2.5 mg^a^	2,109,253	58.52	196,850	5.46	1,040,350	28.86	257,963	7.16	**3,604,415**
Paclitaxel 6 mg^b^	60,776,655	63.34	18,113,116	18.88	5,987,234	6.24	11,082,205	11.55	**95,959,210**
Tamoxifen 20 mg^a^	394,563,360	54.89	205,193,800	28.55	26,791,300	3.73	92,260,920	12.84	**718,809,382**
Trastuzumab 440 mg^b^	3,030,720	10.25	7,537,200	25.49	6,787,880	22.96	12,208,240	41.29	**29,564,040**
Vinorelbine 10 mg^d^	2,058,470	57.79	854,040	23.98	233,700	6.56	415,870	11.67	**3,562,080**
Vinorelbine 20 mg^d^	1,047,080	40.60	1,063,520	41.24	428,380	16.61	40,000	1.55	**2,578,980**
Vinorelbine 30 mg^d^	3,007,350	56.41	1,512,930	28.38	750,600	14.08	60,000	1.13	**5,330,880**

Dosage forms: ^a^tablet; ^b^ampoule; ^c^capsule; ^d^ampoule and capsule.

Source: Integrated General Services Administration System (SIASG)

### Expenditures

Total spending over the period was R$1,012,271,080 reais. The category “Other Institutions” accounted for the largest share of total spending (31.76%), followed by the Ministry of Health (26.24%) (Table 2).

**Table 2:** Public procurement of antineoplastic agents used for treating breast cancer - total spending (R$) by purchaser category. Brazil, 2013–2019

Medicine	Ministry of Education		Ministry of Health		Ministry of Defense		Other Institutions		Total (R$)
	R$	%	R$	%	R$	%	R$	%	
Anastrozole 1 mg^a^	13,196,020	39.67	9,337,267	28.07	5,263,653	15.83	5,464,512	16.43	**33,261,452**
Docetaxel 40 mg^b^	22,886,244	38.63	19,535,557	32.97	6,095,049	10.29	10,729,355	18.11	**59,246,205**
Exemestane 25 mg^c^	16,301,476	50.07	4,857,270	14.92	6,449,958	19.81	4,948,653	15.20	**32,557,358**
Letrozole 2.5 mg^a^	6,683,805	56.11	573,524	4.81	3,549,046	29.79	1,105,916	9.28	**11,912,291**
Paclitaxel 6 mg^b^	26,010,194	57.78	8,515,488	18.92	4,896,219	10.88	5,591,795	12.42	**45,013,696**
Tamoxifen 20 mg^a^	19,865,277	54.79	9,197,183	25.37	1,604,663	4.43	5,589,036	15.42	**36,256,160**
Trastuzumab 440 mg^b^	79,911,241	10.86	195,836,172	26.62	174,182,648	23.68	285,744,659	38.84	**735,674,721**
Vinorelbine 10 mg^d^	6,305,699	57.00	2,153,148	19.46	814,825	7.37	1,788,105	16.16	**11,061,777**
Vinorelbine 20 mg^d^	6,338,358	40.90	6,429,645	41.49	2,498,773	16.13	228,920	1.48	**15,495,696**
Vinorelbine 30 mg^d^	17,960,731	56.49	9,193,434	28.92	4,351,541	13.69	286,020	0.90	**31,791,725**
Total	**215,459,045**	**21.28**	**265,628,688**	**26.24**	**209,706,375**	**20.72**	**321,476,971**	**31.76**	**1,012,271,080**

Dosage forms: ^a^tablet; ^b^ampoule; ^c^capsule; ^d^ampoule and capsule.

Source: Integrated General Services Administration System (SIASG)

**Fig. 1:**
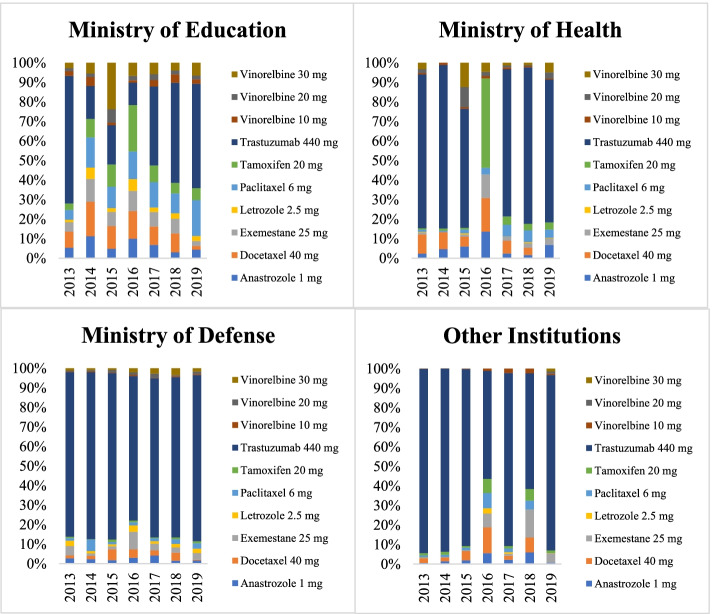
Spending per medicine as a percentage of total spending on antineoplastic agents used for treating breast cancer by purchaser category. Brazil, 2013–2019. Source: Integrated General Services Administration System (SIASG)

The findings show that procurement patterns differ across purchaser categories, with the Ministry of Education showing a considerably different profile to the rest of the categories (Fig. 1). Trastuzumab 440 mg represented the largest share of total spending (72.68%) (Table 2). Spending on trastuzumab as a percentage of overall spending was highest in the categories “Other Institutions” and Ministry of Defense and lowest in the category Ministry of Education. There was a reduction in spending on trastuzumab 440 mg as a percentage of total spending in 2016 across all purchaser categories, with the Ministry of Health not making any purchases of this drug in this year. In contrast, spending on tamoxifen 20 mg as a percentage of total spending increased across all categories. This increase was more pronounced in the category Ministry of Health. The Ministry of Defense was the purchaser category with the most even pattern of spending on these medicines over the study period (Fig. 1).

### Purchasing patterns

**Fig. 2:**
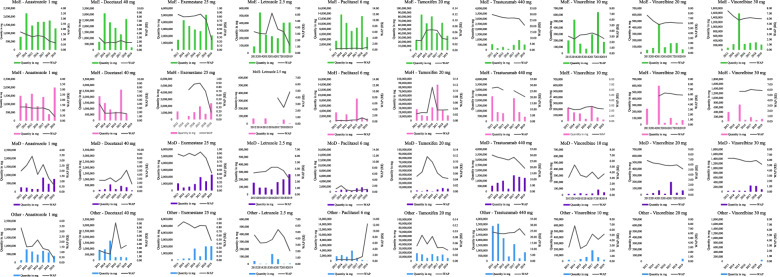
Patterns of weighted average price^a^ per mg (R$) and total purchase quantities of antineoplastic agents used for treating breast cancer by purchaser category. Brazil, 2013–2019. ª Inflation-adjusted annual weighted average prices, compared to December 2019 values. ME: Ministry of Education; MH: Ministry of Health; MD: Ministry of Defense; OI: Other Institutions. Source: Integrated General Services Administration System (SIASG)

There are two main purchasing patterns: an expected or market pattern, where volume of purchases and WAP/mg are inversely proportional; and a pattern characterized by inelasticity, where volume of purchases does not appear to influence WAP/mg. The WAP/mg of trastuzumab 440 mg is apparently inelastic across all purchaser categories throughout the study period, while the WAP/mg of exemestane 25 mg is inelastic in the first three years of the study period across all categories. Other medicines with inelastic WAP/mg include vinorelbine (10, 20 and 30 mg), anastrozole 1 mg and docetaxel 40 mg, which show small variations in price in relation volume purchased by the ministries of health and education. Tamoxifen 20 mg shows a similar pattern across all categories, with prices tending to be higher in 2015 and 2016. It is important to highlight that the scales used for each medicine differ. The findings show price fluctuations over time and differing WAP/mg patterns between medicines and across categories (Fig. 2).

The WAP/mg of paclitaxel 6 mg rose in 2019. This rise was more pronounced in the category “Other Institutions”, probably due to the lower volume of purchases made by this category. In previous years, the WAP/mg of paclitaxel 6 mg was not influenced by volume of purchases across all purchaser categories. Vinorelbine 10 mg, docetaxel 40 mg and anastrozole 1 mg showed different price patterns across different purchaser categories (Fig. 2).

### Avoidable expenditures

Total avoidable expenditure over the study period was R$99,130,645. Avoidable expenditure was highest in the Ministry of Defense and lowest in the category “Other Institutions” (R$32,109,286 and R$13,168,716, respectively). Trastuzumab 440 mg was the medicine with the highest amount of avoidable expenditure in all categories (R$55,428,008). Avoidable expenditure on this drug was highest in the Ministry of Defense (R$21,197,898). Docetaxel 40 mg was the medicine with the second highest amount of avoidable expenditure in the Ministry of Health (R$3,665,684), Ministry of Defense (R$2,925,025), and “Other Institutions” (R$3,034,617). The medicine with the second highest amount of avoidable expenditure in the Ministry of Education was paclitaxel 6 mg (R$3,899,524). The findings show that an additional 23,358,891 units could have been purchased at the lowest WAP/mg, including 11,694,264 units of tamoxifen 20 mg, 5,892,583 units of anastrozole 1 mg, and 4,789,924 units of paclitaxel 6 mg. The number of additional units that could have been purchased was highest in the Ministry of Education (10,440,753) (Table 3).

**Table 3:** Total avoidable expenditure (R$) and additional quantities that could have been purchased at the lowest weighted average price^a^ by medicine (units) and purchaser category. Brazil, 2013–2019

**MINISTRY OF EDUCATION**																
Medicine^&^	**2013**		**2014**		**2015**		**2016**		**2017**		**2018**		**2019**		**Total**	
	**AE (R$)**	**Units**	**AE (R$)**	**Units**	**AE (R$)**	**Units**	**AE (R$)**	**Units**	**AE (R$)**	**Units**	**AE (R$)**	**Units**	**AE (R$)**	**Units**	**AE (R$)**	**Units**
Anastrozole 1 mg^1^	310,500^s^	235,227	350,880^s^	265,818	58,597^s^	48,030	268,443^s^	221,854	83,946^s^	68,249	NA	NA	387,309^o^	1,046,781	1,459,675	1,885,959
Docetaxel 40 mg^2^	390,477^o^	4067	NA	NA	768,635^o^	12,397	NA	NA	552,559^s^	7467	391,184^s^	5927	10,923^d^	155	2,113,778	30,014
Exemestane 25 mg^3^	260,230^s^	15,536	NA	NA	241,621^s^	13,240	117,260^o^	6013	27,111^o^	1276	101,956^s^	5826	126,217^s^	28,048	874,396	69,939
Letrozole 2.5 mg^1^	NA	NA	NA	NA	80,412^s^	12,092	539,006^o^	56,001	NA	NA	340,783^s^	54,525	NA	NA	960,201	122,618
Paclitaxel 6 mg^2^	301,885^s^	173,497	NA	NA	1,672,640^s^	1,161,556	712,742^d^	329,973	NA	NA	NA	NA	1,212,257^s^	396,162	3,899,524	2,061,188
Tamoxifen 20 mg^1^	271,405^s^	678,513	NA	NA	2,451,137^s^	4,085,228	NA	NA	1,158,080^s^	1,447,600	NA	NA	NA	NA	3,880,623	6,211,342
Trastuzumab 440 mg^2^	3,131,527^o^	295	920,266^o^	90	1,304,827^o^	131	328,739^o^	32	1,506,941^o^	144	3,185,909^s^	315	843,025^d^	100	11,221,235	1107
Vinorelbine 10 mg^4^	97,010^s^	3789	188,306^s^	8559	153,513^s^	6823	48,856^s^	1816	230,746^s^	8125	506,126^s^	20,574	49,657^s^	2104	1,274,214	51,791
Vinorelbine 20 mg^4^	41,334^d^	304	14,394^d^	114	27,455^s^	242	20,011^d^	170	61,538^d^	531	21,901^s^	185	51,889^d^	504	238,522	2050
Vinorelbine 30 mg^4^	107,430^d^	567	41,584^d^	219	63,109^s^	370	92,181^s^	539	148,594^d^	869	48,123^d^	271	273,096^o^	1908	774,117	4744
**MINISTRY OF HEALTH**																
Medicine^&^	**2013**		**2014**		**2015**		**2016**		**2017**		**2018**		**2019**		**Total**	
	**AE (R$)**	**Units**	**AE (R$)**	**Units**	**AE (R$)**	**Units**	**AE (R$)**	**Units**	**AE (R$)**	**Units**	**AE (R$)**	**Units**	**AE (R$)**	**Units**	**AE (R$)**	**Units**
Anastrozole 1 mg^1^	NA	NA	NA	NA	NA	NA	NA	NA	NA	NA	82,660^e^	99,591	99,310^o^	268,405	181,970	367,995
Docetaxel 40 mg^2^	3,247,794^o^	33,831	147,224^e^	2217	206,006^o^	3323	64,661^e^	913	NA	NA	NA	NA	NA	NA	3,665,685	40,284
Exemestane 25 mg^3^	NA	NA	NA	NA	NA	NA	96,606^o^	4954	96,196^o^	4527	NA	NA	NA	NA	192,801	9481
Letrozole 2.5 mg^1^	1362^e^	164	NA	NA	NA	NA	NA	NA	230^e^	25	NA	NA	16,162^e^	5213	17,753	5402
Paclitaxel 6 mg^2^	NA	NA	5711^e^	2884	NA	NA	61,613^d^	28,525	171,549^e^	58,350	1,068,095^e^	468,463	NA	NA	1,306,968	558,222
Tamoxifen 20 mg^1^	NA	NA	NA	NA	NA	NA	1,285,271^e^	918,051	NA	NA	NA	NA	NA	NA	1,285,271	918,051
Trastuzumab 440 mg^2^	8,496,516^o^	801	4,553,299^o^	447	2,594,719^o^	260	NA	NA	3,746,929^o^	358	NA	NA	570,309^d^	67	19,961,772	1934
Vinorelbine 10 mg^4^	NA	NA	NA	NA	NA	NA	NA	NA	NA	NA	NA	NA	NA	NA	NA	NA
Vinorelbine 20 mg^4^	31,586^d^	232	NA	NA	NA	NA	10,337^d^	88	36,843^d^	318	NA	NA	48,881^d^	475	127,648	1113
Vinorelbine 30 mg^4^	240,228^d^	1267	NA	NA	NA	NA	NA	NA	62,664^d^	366	1021^d^	6	112,578^o^	787	416,490	2426
**MINISTRY OF DEFENSE**																
Medicine^&^	**2013**		**2014**		**2015**		**2016**		**2017**		**2018**		**2019**		**Total**	
	**AE (R$)**	**Units**	**AE (R$)**	**Units**	**AE (R$)**	**Units**	**AE (R$)**	**Units**	**AE (R$)**	**Units**	**AE (R$)**	**Units**	**AE (R$)**	**Units**	**AE (R$)**	**Units**
Anastrozole 1 mg^1^	175,371^s^	132,857	301,078^s^	228,090	364,487^s^	298,759	71,568^s^	59,147	1,160,270^s^	943,309	229,449^e^	276,445	185,286^o^	500,773	2,487,509	2,439,380
Docetaxel 40 mg^2^	28,038^o^	292	159,722^e^	2405	945,109^o^	15,244	81,561^e^	1152	592,045^s^	8001	1,118,551^s^	16,948	NA	NA	2,925,026	44,042
Exemestane 25 mg^3^	219,671^s^	13,115	13,113^e^	656	96,936^s^	5312	65,326^o^	3350	156,005^o^	7341	121,042^s^	6917	572,184^s^	127,152	1,244,278	163,842
Letrozole 2.5 mg^1^	4687^e^	563	40,085^e^	5257	82,559^s^	12,415	26,446^o^	2748	83,644^e^	8946	244,233^s^	39,077	318,416^e^	102,715	800,071	171,721
Paclitaxel 6 mg^2^	193,477^s^	111,194	1,335,805^e^	674,649	249,584^s^	173,322	NA	NA	79,445^e^	27,022	325,684^e^	142,844	337,189^s^	110,193	2,521,184	1,239,223
Tamoxifen 20 mg^1^	72,358^s^	180,896	31,192^s^	51,986	295,977^s^	493,295	22,452^e^	16,037	97,743^s^	122,179	76,082^s^	95,103	NA	NA	595,805	959,496
Trastuzumab 440 mg^2^	3,269,234^o^	308	4,268,039^o^	419	4,736,421^o^	475	639,984^o^	62	6,876,263^o^	657	1,407,957^s^	139	NA	NA	21,197,898	2060
Vinorelbine 10 mg^4^	9412^s^	368	65,409^s^	2973	36,320^s^	1614	8213^s^	305	30,110^s^	1060	33,388^s^	1357	57,289^s^	2427	240,141	10,105
Vinorelbine 20 mg^4^	NA	NA	NA	NA	33,911^s^	299	NA	NA	NA	NA	323^s^	3	NA	NA	34,234	301
Vinorelbine 30 mg^4^	NA	NA	NA	NA	19,258^s^	113	2632^s^	15	NA	NA	NA	NA	41,251^o^	288	63,141	417
**OTHER INSTITUTIONS**																
Medicine^&^	**2013**		**2014**		**2015**		**2016**		**2017**		**2018**		**2019**		**Total**	
	**AE (R$)**	**Units**	**AE (R$)**	**Units**	**AE (R$)**	**Units**	**AE (R$)**	**Units**	**AE (R$)**	**Units**	**AE (R$)**	**Units**	**AE (R$)**	**Units**	**AE (R$)**	**Units**
Anastrozole 1 mg^1^	273,649^s^	207,310	148,643^s^	112,608	215,818^s^	176,900	456,011^s^	376,868	76,409^s^	62,121	218,657^e^	263,442	NA	NA	1,389,186	1,199,249
Docetaxel 40 mg^2^	NA	NA	213,948^e^	3222	NA	NA	2,007,159^e^	28,350	322,482^s^	4358	491,028^s^	7440	NA	NA	3,034,617	43,369
Exemestane 25 mg^3^	15,042^s^	898	28,088^e^	1404	36,445^s^	1997	NA	NA	NA	NA	280,207^s^	16,012	610,704^s^	135,712	970,486	156,023
Letrozole 2.5 mg^1^	49,663^e^	5966	1082^e^	142	3500^s^	526	NA	NA	108,797^e^	11,636	4065^s^	650	5913^e^	1907	173,020	20,827
Paclitaxel 6 mg^2^	411,748^s^	236,637	245,363^e^	123,921	362,000^s^	251,389	39,448^d^	18,263	263,379^e^	89,585	404,387^e^	177,363	104,450^s^	34,134	1,830,775	931,291
Tamoxifen 20 mg^1^	568,902^s^	1,422,254	918,086^s^	1,530,144	213,870^s^	356,450	168,534^e^	120,382	NA	NA	140,916^s^	176,146	NA	NA	2,010,309	3,605,375
Trastuzumab 440 mg^2^	NA	NA	NA	NA	NA	NA	NA	NA	NA	NA	1,050,590^s^	104	1,996,514^d^	236	3,047,104	340
Vinorelbine 10 mg^4^	3422^s^	134	34,022^s^	1546	7198^s^	320	28,195^s^	1048	428,934^s^	15,103	114,889^s^	4670	73,749^s^	3125	690,408	25,947
Vinorelbine 20 mg^4^	NA	NA	NA	NA	NA	NA	NA	NA	NA	NA	NA	NA	22,812^d^	221	22,812	221
Vinorelbine 30 mg^4^	NA	NA	NA	NA	NA	NA	NA	NA	NA	NA	NA	NA	NA	NA	NA	NA

Legend: *AE* avoidable expenditure. Units – number of units. *NA* not applicable. ^e^ Ministry of Education, ^s^ Ministry of Health, ^d^ Ministry of Defense, ^o^ Other Institutions

ª Inflation-adjusted annual weighted average prices, compared to December 2019 values

Dosage forms: ^1^tablet; ^2^ampoule; ^3^capsule; ^4^ampoule and capsule

Source: Integrated General Services Administration System (SIASG)

## Discussion

The findings show that the Ministry of Education accounted for the largest volume of antineoplastic agent purchases during the study period. This can be explained by the large number of purchases made by complex-care university hospitals, which play an important role in cancer care, education and research in the country [16, 17]. Total spending on antineoplastic drugs during the study period was more than R$1 billion. According to the literature, the rising cost of antineoplastic drugs may be caused by a number of different factors, including: increased access and utilization [18, 19] (due to growing incidence of different types of cancer and wider access to diagnosis and, consequently, treatment); an increase in prices [20, 21] of both newly-approved technologies and medicines already firmly established on the market; and changes in drug utilization profiles [22, 23], including an increase in purchases of higher-cost medicines fuelled by the rising number of patients needing treatment for advanced cancer. However, despite the sharp rise in spending on antineoplastic agents, the availability of these medicines remains low in many countries, including Brazil [24–26].

The largest component of breast cancer patient costs is systemic therapy, which includes chemotherapy and hormone therapy [27, 28]. Anticancer therapy has a high cost for the SUS. A study conducted by Lana [29] showed that the SUS spent R$14.9 billion on cancer care between 2001 and 2014, with the treatment of breast cancer accounting for the largest share of expenditure (R$6.4 billion or 43% of spending on the cancers investigated by the study). In addition, chemotherapy represented the largest share of direct costs associated with all types of cancer analyzed by the study (R$9538.7 million, equivalent to 64% of total cancer care costs). Data show that total direct expenditure on admissions, chemotherapy and social security benefits for people with breast cancer rose by 110% between 2008 and 2015, from approximately R$302 million to R$633 million, with chemotherapy accounting for 68% of total spending [30]. In 2018, antineoplastic agents and immunomodulators led sales (16.4% of overall drug purchases), amounting to more than R$12.4 billion [31].

The purchaser category that spent most on antineoplastic agents over the study period was “Other Institutions”, followed by the Ministry of Health. It is important to highlight that state and municipal health authorities represent a significant share of the procuring entities in the category “Other Institutions”. In a public interest civil action brought by the public prosecutor’s office in the Federal District, it was found that certain companies charged a state health authority different prices than those already agreed in contracts to supply medicines awarded by the Ministry of Health [32]. This may be explained by the law of supply and demand, bearing in mind that state and municipal health authorities tend make considerably smaller purchases than the Ministry of Health.

The findings show that the purchasing patterns observed in the Ministry of Education differ from those of the other purchaser categories. This may be explained by a number of factors, including: the variety of possible treatments for the same type of tumor; adoption of different protocols (due to the lack of clinical protocols and the flexibility of DDTs, meaning that service providers can choose what treatment to use) [33]; use of medicines in research and clinical trials [17]; different approaches to hospital management^15^; and non-centralized purchases [34]. There was a notable change in the distribution of expenditure in 2016. Spending on trastuzumab 440 mg as a percentage of overall spending decreased across all purchaser categories, with the Ministry of Health not making any purchases of this drug in this year. For want of an explanation in the literature, it is assumed that this reduction is associated with a number of factors, including stockpiling, supply problems, and purchasing difficulties and/or budget shortfalls. However, the fall in spending on trastuzumab as a percentage of overall spending does not appear to be related to a reduction in utilization. In this regard, a study by Ferraris [35] analyzing the utilization of trastuzumab for the treatment of breast cancer in the state of Rio de Janeiro observed that services surpassed the number of expected procedures in 2016.

Our findings also show that the response of WAP/mg to quantity procured differs according to drug and purchaser category. WAP/mg and demand were shown to be both elastic and inelastic. The literature shows that medicines, especially antineoplastic agents, have peculiar market characteristics – such as lack of supply chain transparency, limited competition due to market segmentation and monopolies over medicines, and imbalances between supply, consumption and demand – which can contribute to price variations across regions and countries [36, 37].

Trastuzumab accounted for the largest share of total spending across all purchaser categories. The category “Other Institutions” led purchases of this drug, representing around 40% of total spending, corroborating the findings of Moraes et al. [34], who investigated the potential implications of global trastuzumab price policies in seven countries in Latina America. The study shows that the medicine was considered unprofitable in 2015, meaning that it was necessary to cut prices by between 70 and 95% to make it cost effective [38]. In this regard, it is known that high drug prices and high general treatment costs in poorly structured health systems are barriers to access [18, 39].

While trastuzumab was the country’s top-selling active ingredient in terms of revenue in 2018 [31], access to high-cost medicines on the SUS is restricted and generally well behind developed countries when it comes to newly-approved medicines [19, 25]. Trastuzumab is also notable for its price inelasticity of demand across all purchaser categories. This may be explained by the complexity and structure of the market for antineoplastic drugs and other factors that make medicine prices less predictable [1]. The drug that accounted for the largest volume of purchases was tamoxifen. This may be explained by the fact that tamoxifen is the most commonly used medicine in hormonal therapy (indicated for the treatment of early and advanced-stage breast cancer in pre and post-menopausal women), being standard treatment by consensus and according to clinical guidelines and associated with gains in *disease*-*free* and *overall survival* [40, 41].

From a cost-effectiveness perspective, the findings show that total avoidable expenditure was approximately R$100 million. It is known that underfunding and irregular cash flow have always been inherent problems in Brazil’s public health system [42]. In this regard, this study reveals that procurement problems extend beyond budget deficits to include poor management and high WAP/mg. The data show that the potential savings made by purchasing at the lowest WAP/mg could have been used to purchase a significant quantity of medicines, expanding availability and consequently enabling wider access to antineoplastic drugs on the SUS. This is particularly relevant given that the SUS has limited facilities to absorb breast cancer patient demand and provided adequate treatment [43], including deficiencies in screening and diagnosis, consequently leading to delays in various stages of treatment and contributing to negative prognoses [44].

The Ministry of Defense was the poorest performing purchaser category when it comes to WAP/mg and consequently responsible for the highest amount of avoidable expenditure during the study period. A study by Moraes et al. [34] analyzing federal government procurement of the antineoplastic drugs imatinib mesylate, trastuzumab and L-asparaginase also reported that the procuring entity that paid the highest mean prices was the Ministry of Defense. The medicine that accounted for the largest share of avoidable expenditure was trastuzumab, followed by docetaxel, except in the Ministry of Education, where paclitaxel represented the second largest share. The findings show that by purchasing at the lowest WAP/mg it would have been possible to acquire an additional 11,694,264 units of tamoxifen 20 mg, 5,892,583 units of anastrozole 1 mg and 4,789,924 units of paclitaxel 6 mg. This shows the importance of analyzing avoidable expenditure, especially given the effects of cancer patient treatment costs on society, the government and health systems [45]. The importance of assessing avoidable expenditure is reinforced when we look at the number of additional units that could have been purchased as a percentage of the total quantities of each medicine purchased over the study period: tamoxifen (32.54%), paclitaxel 6 mg (30%), vinorelbine 10 mg (24.66%) and docetaxel (23.73%). These additional units could have played an important role in reducing iniquity in access to chemotherapy drugs provided by the SUS.

The present study analyzed public procurement using competitive bidding procedures, excluding drug purchases by court order. However, given the increased judicialization of purchases as an alternative means to expand access to antineoplastic agents through the SUS, it is important to highlight that the cost of bringing such lawsuits is high, constituting another factor that contributes to the growing economic burden of cancer [46, 47]. Effective procurement would avoid unnecessary expense and help increase the availability of medicines on the SUS, consequently minimizing spending on health litigation.

This study has some limitations. First, the exclusion of medicines that were not purchased by the Ministry of Health in at least five of the seven years of the study period meant that a complete overview of adjuvant and neoadjuvant therapy for breast cancer was unobtainable. Second, some of the antineoplastic agents investigated by this study are used for treating diseases other than breast cancer which may overrepresent consumption for breast cancer. However, this limitation is partially overcome because in the Brazilian public system prescribing of these medicines is restricted to cancer treatments. While this may broaden actual use to other cancer indications, all would profit from elimination of avoidable expenditures. Finally, SIASG is a national aggregated database of drug purchases by procuring entities and does not include individually prescribed/dispensed medicines, meaning it is not possible to identify therapeutic indication.

## Conclusions

The Brazilian Ministry of Education was the largest purchaser of antineoplastic agents in terms of volume, showing considerably different spending patterns to the rest of the purchaser categories. State and municipal health authorities spent most on antineoplastic agents, mainly with trastuzumab. Tamoxifen and trastuzumab accounted for the largest volume of purchases and largest share of total spending, respectively. Tamoxifen is considered the gold standard for breast cancer treatment while trastuzumab was characterized by price inelasticity of demand. Total avoidable expenditure was approximately R$100 million and overspending was highest in the Ministry of Defense.

Breast cancer is the most common cancer in women in Brazil and studying public procurement of antineoplastic agents used for the treatment of this disease is of utmost importance. The findings also suggest that the SUS is facing a shortage of medicines in high-complexity health care facilities and high-complexity cancer care centers. The effective management of public procurement of antineoplastic agents can help expand access to these medicines and promote the financial sustainability of the SUS.

## Abbreviations

AC: Doxorubicin (adriamycin), Cyclophosphamide
ATC: Anatomical Therapeutic Chemical
CAF: Cyclophosphamide, Doxorubicin, 5-fluorouracil
CEF: Cyclophosphamide, Epirubicin, 5-fluorouracil
CMF: Cyclophosphamide, Methotrexate, 5-fluorouracil
DC: Docetaxel, Cyclophosphamide
DDD: Defined Daily Dose
INCA: National Cancer Institute
IPCA: National Consumer Price Index
SIASG: Integrated General Services Administration System
SUS: *Sistema Único de Saúde*
WAP: Weighted Average Price
WAP/mg: Weighted Average Price per mg
WHO: World Health Organization

## Footnotes

1 The following agents were excluded because they were not bought by the Ministry of Health in at least five of the seven years of the study period: docetaxel 20 mg, paclitaxel 100 mg, tamoxifen 10 mg, trastuzumab 120 and 150 mg, and vinorelbine 40 and 80 mg.
